# The Effect and Mechanism of Curcumin Combined with Carboplatin Chemotherapy Promoting on Apoptosis of Lung Cancer HCC827 Cells

**DOI:** 10.1155/2022/1932692

**Published:** 2022-08-08

**Authors:** Lingping Huang, Xiangyuan He, Xuan Zuo

**Affiliations:** ^1^Department of Public Health and Preventive Medicine, Medical School, Jinggangshan University, China; ^2^Thoracic Surgery, First Affiliated Hospital of Nanchang University, China; ^3^Department of Respiratory Medicine, Affiliated Hospital of Jinggangshan University, China

## Abstract

**Objective:**

To investigate the effect and mechanism of curcumin (CUR) killing lung cancer HCC827 cell spheres.

**Method:**

HCC827 cell spheres were cultured in serum-free medium, and the protein expression of CD133, SOX2, EpCAM, and ABCG2 was detected by western blot. MTT was used to evaluate the cell viability of HCC827 cell spheres and HCC827 cell after they were treated by 1, 2, 5, 10, and 20 mg/mL carboplatin (CBP) for 48 h. The inhibitory effects of 10 *μ*M, 50 *μ*M, 100 *μ*M, and 200 *μ*M CUR on GST (glutathione S-transferase) activity in HCC827 cell spheres were determined by colorimetry. The flow cytometry (FCM), western blot, qPCR, luciferase assay, and microscopy were used to detect the ROS levels, cell pelletization ability, *β*-catenin, SOX2, and ABCG2 mRNA and the promoter activity of *β*-catenin upon of HCC827 cell spheres treated with 200 *μ*M CUR for 48 h. The HCC827 cell spheres were infected with *β*-catenin adenovirus, and then cells were treated with 200 *μ*M CUR (and/or no 5 mg/mL CBP) for 24 h. The mRNA and protein expression of *β*-catenin, SOX2, and ABCG2 was detected by qPCR and western blot, and cell growth inhibition of HCC827 cell spheres was evaluated by MTT.

**Result:**

The expression of stem cells marker CD133, SOX2, EpCAM, and drug resistance-related gene ABCG2 mRNA is higher in HCC827 cell spheres, and HCC827 cell spheres resisted the killing effect of difference doses of CBP. The activity of GST of HCC827 cell spheres was inhibited by 10 *μ*M, 50 *μ*M, 100 *μ*M, and 200 *μ*M CUR. It was a dose-dependent manner. After 200 *μ*M CUR had treated HCC827 cell spheres for 48 h, the level of ROS was significantly increased (*P* < 0.05), and the mRNA and protein expression of *β*-catenin, SOX2, and ABCG2 and promoter activity of *β*-catenin were notably decreased (*P* < 0.05), compared to the control group. Furthermore, the formed-sphere ability of HCC827 sphere was inhibited after cells were treated with 200 *μ*M CUR. 200 *μ*M CUR could suppress the proliferation of HCC827 cell spheres and induced cell apoptosis. The proliferation of HCC827 cell spheres was significantly inhibited, and cell apoptosis rate was increased by 200 *μ*M CUR combined with 5 mg/mL CBP than by 200 *μ*M CUR alone. Upregulation of *β*-catenin by adenovirus partly reversed the effect of CUR inhibition of the expression of *β*-catenin, SOX2, and ABCG2, compared to empty vector adenovirus group. Additionally, overexpression of *β*-catenin significantly remitted the inhibitory effect of 200 *μ*M CUR combined with 5 mg/mL CBP on the proliferation of HCC827 cell spheres.

**Conclusion:**

CUR inhibited the cell proliferation and stem cell trait and induced apoptosis in HCC827 cell spheres by the inhibition of GST activity and *β*-catenin expression. CUR is expected to become a treatment for lung cancer and lung cancer stem cells.

## 1. Introduction

Cancer stem cells (CSCs) are a group of tumor cells with unlimited self-renewal, multidirectional differentiation ability, and stronger proliferation ability [1, 2]. A large number of studies have shown that lung cancer cells contain a small population of cells. After isolation and culture, it is found that in addition to self-renewal and differentiation ability, its proliferation ability is stronger than ordinary lung cancer cells, and it has natural tolerance to chemotherapy drugs [3–5]. This population of cells is called lung cancer stem cells (LCSCs). Since it is difficult to kill LCSCs with current first-line chemotherapy drugs (such as carboplatin), LCSCs become the seeds of recurrence, drug resistance, and metastasis of lung cancer after chemotherapy [6]. Therefore, exploring new strategies to reverse LCSCs resistance is a hot research topic at present.

Glutathione S-transferase (GST) is a group of phase sugar-metabolizing enzymes ubiquitous in various organisms, which play an important role in the metabolism and detoxification of many endogenous and exogenous substances, including environmental carcinogens, chemotherapeutic drugs, and reactive oxygen species. Inhibition of GST activity is an effective therapy for many cancers. So far, many kinds of GST (glutathione S-transferase) inhibitors have been found, mainly including some ketones such as complexes of ethanoic acid, malonaldehyde, cinnamaldehyde, curcumin, and glutathione [7–10]. Curcumin has been studied as a potential inhibitor of human recombinant GSTA1, GSTM1, and GSTP1 [11, 12]. Curcumin (diindolylmethane) is a natural compound isolated from the plant turmeric. Its main composition is 2-5% turmeric [13]. Mukherjee et al. found that curcumin could be a good candidate for the treatment of NSCLC [14]. In addition, researchers have found that CUR increases levels of oxidative stress in tumor cells and induces cell death [15]. Curcumin has potential as a chemical sensitizer for carboplatin in NSCLC patients, and curcumin in combination with carboplatin can inhibit antitumor activity [16]. In this study, we obtained HCC827 cell spheres rich in lung cancer stem cells to study the influence of CUR on the drug resistance of LCSCs and the killing effect and mechanism of HCC827 cell spheres.

## 2. Materials and Methods

### 2.1. Reagents

The following reagents are used: F12K medium, DMEM/F12 medium and B27(50×) (Gibco, USA); recombinant human epithelial growth factor (rhEGF) (Protech, USA); CUR, insulin, and basic fibroblast growth factor (bFGF) (Sigma, USA); Fetal Bovine Serum (FBS) (Pan, Australia); antibody CD133, EpCAM, SOX2, ABCG2, and *β*-catenin (Abcam, USA); total protein lysis reagent, *β*-actin antibody and HRP labeled secondary antibody, reactive oxygen species (ROS) kits, and CCK-8 kits (Beyotime, Shanghai); Electronics Components Laboratory (ECL) (Millipore, Shanghai); GST Activity Detection Kit (BioVision, USA); Annexin V-FITC Kit (KeyGEN BioTECH, Jiangsu); RT-PCR Kit and qRT-PCR Kit; (Thermo, USA); and the luc-*β*-catenin and luc-TK adenovirus and *β*-catenin overexpressed adenovirus (Hanbio, Shanghai).

### 2.2. Cell Culture

HCC827 cells were cultured with F12K+10% fetal bovine serum, and cells at logarithmic growth stage were selected for the next experiment. HCC827 cells were inoculated into 6-well plates with 200 cells/well. After the cells grew into clones (about 10-14 d), the holoclone cells were digested by the clone ring. The cells were placed in the ultralow adhesion culture plate containing SFM for culture, and an appropriate amount of medium was added to each well of the 6-well plate every 3 days. After 2 weeks, the cells were observed to form multicellular spheres suspended in the medium, and the multicellular spheres were collected and digested in trypsin. The cell spheres were blown to become single cells, and after centrifugation and washing, they were inoculated in SFM for further culture, and the second generation of multicell spheres were expanded to form and relevant experiments were conducted. This experiment has been approved by the Ethics Committee of the Department of Jinggangshan University.

### 2.3. Experiment Treatment

HCC827 cells and HCC827 cell spheres were treated with CBP. The HCC827 cells and HCC827 cell spheres were digested and inoculated into culture flask or culture plate for 24 h. HCC827 and cell spheres were treated with CBP (HANSOH PHARMA, Jiangsu) at concentrations of 1, 2, 5, 10, and 20 mg/mL for 48 h; HCC827 cell spheres were treated with CUR. The HCC827 cell spheres are digested and placed in the culture for 24 h. The nutrient solution is replaced with fresh SFM medium, and various doses of CUR were added for 48 h.

### 2.4. Determination of GST Activity

The treated cells were scraped off and collected with a cell scraper. The cells were washed once with PBS, and 200 *μ*L GST Assay Buffer was added. The cells were broken by an ultrasonic breaker, centrifuged at 4°C for 12000 RPM for 10 min, and the supernatant was collected for detection. After 50 *μ*L of samples, positive and negative controls were taken, remixed with 5 *μ*L of glutathione and 1 *μ*L GST reaction substrate (CDNB) at room temperature for 5 min; the absorbance was measured.

### 2.5. DCFH-DA Detected Intracellular Reactive Oxygen Species (ROS)

After 2 × 10^7^ cells/mL were collected, they were suspended in dCFH-DA working solution diluted with 1 mL serum-free medium (1 : 1000) and incubated in 37°C cell culture chamber for 20 min. The mix was reversed every 3–5 min to fully contact the probe and cells. The cells were washed three times with serum-free cell culture medium to fully remove DCFH-DA that did not enter the cells. The cells were diluted with 200 *μ*L special Buffer and detected by flow cytometry with excitation wavelength of OD_488nm_.

### 2.6. Apoptosis Rate Was Detected by FITC-Annexin V/PI Staining

After 2 × 10^7^ cells/mL were collected and resuspended with PBS (buffer) containing 2% FBS. The supernatant was discarded after centrifugation for 10 min. Annexin V-FITC (5 *μ*L) was added with 80 *μ*L buffer and mixed with Annexin V-FITC. Add 5 *μ*L PI staining and incubate at room temperature away from light for 10 min. Flow cytometry was used for detection.

### 2.7. *β*-Catenin Promoter Activity Analysis

The HCC827 cell spheres were digested into cell suspension, and the cell concentration was adjusted to 1 × 10^5^/well after counting, and the cells were inoculated on 6-well plates. After 24 h culture in SFM medium, HCC827 cell spheres were infected with luc-*β*-catenin adenovirus (MOI = 200) for 12 h and then TK adenovirus (as internal reference) for 12 h. After replacement with fresh SFM medium, CUR of 200 *μ*M was added for 24 h. Then, wash the cells with PBS, add Passive Lysis Buffer, and transfer the lysis solution to the 96-well plates. Add luciferase assay reagent for luciferase fluorescence. After mixing with Stop Reagent, the relative *β*-catenin promoter activity was detected for luciferase fluorescence of the control sea kidney.

### 2.8. Adenovirus *β*-Catenin (Adv. *β*-Catenin) Infects HCC827 Cell Globules

The HCC827 cell spheres were digested into cell suspension and inoculated in petri dishes. After 24 h culture, Adv. *β*-catenin (MOI = 200) was added into the fresh antibiotic free SFM medium for another 12 h, and then, the culture was changed to the next step.

### 2.9. CCK-8 Examined as well as HCC827 and Survival of Cell Spheres after Different Treatments

HCC827 and HCC827 cell spheres with good growth were digested into cell suspension. The cells were counted, and the cell concentration was adjusted to 3 × 10^3^ cells/mL. The cells were inoculated on 96-well plates and then added with F12K and SFM culture medium for treatment. Add 10 *μ*L CCK-8 solution to each well at 12 h, 24 h, 36 h, and 48 h, respectively, and incubate for 4 h. The absorbance was measured at OD_570nm_ by enzyme-linked immunosorbent assay.

### 2.10. Expression of *β*-Catenin, SOX2, and ABCG2 Was Detected by RT-PCR

After HCC827 cell spheres were collected, total RNA was extracted with TRIzol. Total RNA was reversely transcribed into cDNA using a reverse transcription kit. The expression of *β*-catenin, SOX2, and ABCG2 mRNA was detected by real-time fluorescence quantitative PCR using Bio-rad CFX Manager system, with *β*-actin as the internal reference (Table 1). Each gene to be tested was set with 3 multiple pores. According to the formula RQ = 2^−△△CT^, the multiple relationship between each group was calculated.

### 2.11. Expression of the CD133, EpCAM, SOX2, ABCG2, and Catenin Proteins Was Determined by Western Blot

After direct centrifugation of the suspended cell pellet, 300 L of protein lysate and protease inhibitor mixture was added and lysed on ice for 10 min. The adherent cells were digested with trypsin, centrifuged, collected, and lysed. The supernatant was centrifuged at 12000 r/min at 4°C for 20 min. The total protein was denatured by SDS-PAGE electrophoresis. After wetting the membrane, the PVDF membrane was sealed at room temperature for 1 h, and the antibody diluent of CD133, EpCAM, SOX2, ABCG2, and *β*-catenin (BSA dilution ratio of 1 : 500) and rabbit anti-*β*-actin antibody (dilution ratio of 1 : 10000) was added, respectively, and incubated at 4°C overnight. After washing TBST, the secondary antibody was incubated at room temperature for 1 h. The expression levels of various proteins were detected by the ECL chemiluminescence method. Images of protein bands were analyzed using Image-Pro Plus 10.0, and the same sample *β*-actin was used as the internal reference to correct the target proteins.

### 2.12. Statistical Analysis

Each experiment was repeated at least 3 times. SPSS 13.0 was used to analyze the data. A *t*-test was performed between groups, and one-way ANOVA was performed within groups. *P* < 0.05 was considered statistically significant.

## 3. Results

### 3.1. Culture of HCC827 Cell Spheres, Stem Cell Markers, and Drug Resistance Detection

**Figure 1:**
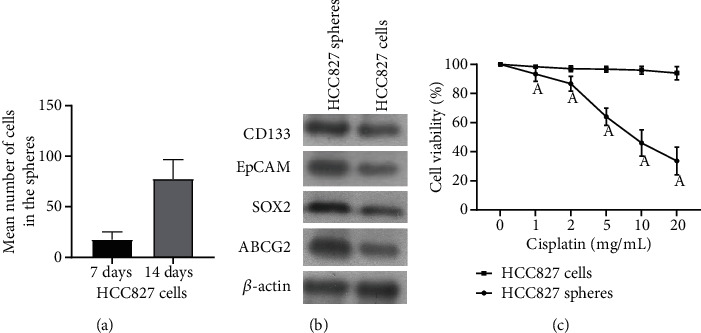
Expression and drug resistance of stem cell markers in HCC827 cell spheres. (a) HCC827 cell cloning under phase contrast microscope (×200). (b) Western blot analysis of the expression levels of stem cell markers CD133, EpCAM, SOX2, and ABCG2 in HCC827 cell spheres and HCC827 cells. (c) MTT was used to detect the cell viability of HCC827 cells and HCC827 cell spheres treated with different CBP for 48 h; ^A^compared with the HCC827 cell group (*P* < 0.05).

After HCC827 cells were cultured for 7 days, holoclone and paraclone clones could be seen. After the holoclone was cultured with SFM for 14 days using the clone ring, 50-100 cells could be observed to gather and form cell spheres suspended in the medium with strong refraction (Figure 1(a)). The expression levels of CD133, EpCAM, SOX2, and ABCG2 were significantly higher in HCC827 cell spheres than in HCC827 cells (Figure 1(b)). HCC827 cell spheres and HCC827 cells were treated with different concentrations of CBP. The cell viability of HCC827 cells was significantly downregulated with the increase of concentration (*P* < 0.05), while the cell viability of HCC827 cells had no significant change (*P* > 0.05) (Figure 1(c)).

### 3.2. Effects of CUR on GST Activity and ROS Levels in HCC827 Cell Spheres

**Figure 2:**
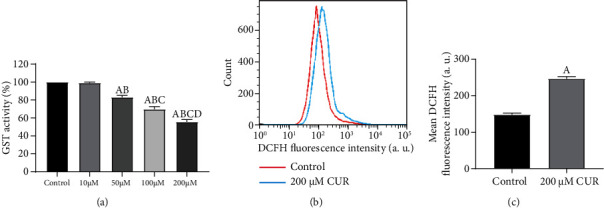
CUR inhibits GST activity in HCC827 cell spheres and upregulates intracellular ROS level. (a) Changes of GST activity in HCC827 cell spheres treated with CUR at different concentrations for 48 h. ^A^Compared with the control group, *P* < 0.05; ^B^compared with the 10 *μ*M CUR group, *P* < 0.05; ^C^compared with the 50 *μ*M CUR group, *P* < 0.05; ^D^compared with the 100 *μ*M CUR group, *P* < 0.05. (b) Flow cytometry was used to detect the fluorescence intensity of DCFH after HCC827 cell spheres were treated with 200 *μ*M CUR for 48 h (intracellular ROS level). (c) The fluorescence intensity of DCFH in different treatment groups was analyzed statistically. ^A^Compared with the control group, *P* < 0.05.

With the CUR concentration increasing from 10 *μ*M to 200 *μ*M, the activity of GST in HCC827 cell spheres decreased gradually (Figure 2(a)) (*P* < 0.05). Since 200 *μ*M CUR treatment reduced the activity of GST to a minimum, the concentration of 200 *μ*M was used to treat HCC827 cell spheres in the subsequent experiments. ROS levels were detected by flow cytometry after HCC827 cell spheres were treated with 200 *μ*M CUR for 48 h (Figures 2(b) and 2(c)). The ROS level of CUR treatment group was significantly higher than that of control group (*P* < 0.01). These results indicate that CUR can specifically inhibit GST activity and upregulate ROS level.

### 3.3. CUR Inhibited the Growth of HCC827 Cell Spheres and Promoted Apoptosis

**Figure 3:**
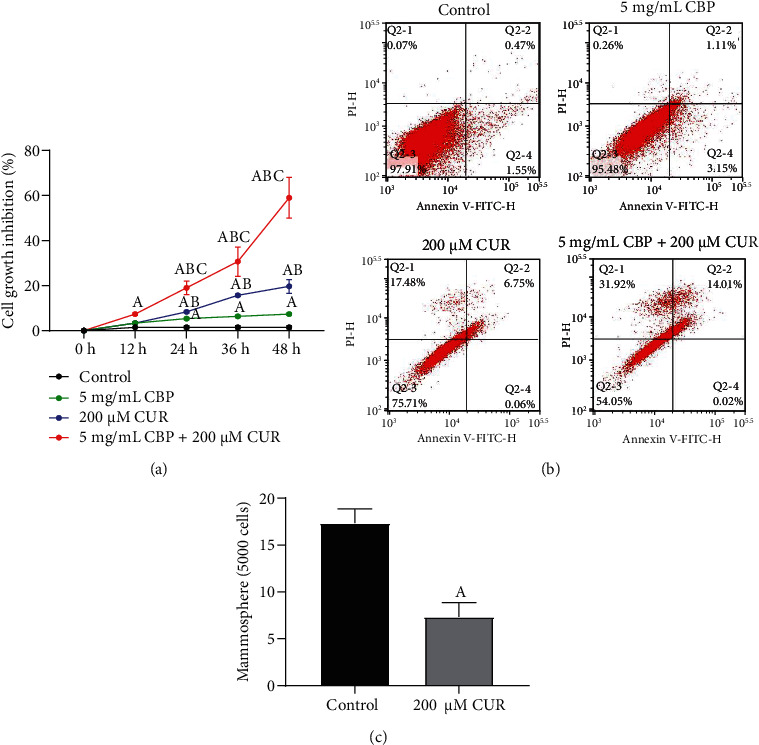
CUR inhibited the growth of HCC827 cell spheres and promoted apoptosis. (a) CCK-8 detected the change of inhibition rate of HCC827 cell spheres treated with 200 *μ*M CUR, 5 mg/mL CBP, and their combination for 48 h. ^A^Compared with the control group, *P* < 0.05; ^B^compared with 5 mg/mL CBP group, *P* < 0.05; ^C^compared with the 200 *μ*M CUR group, *P* < 0.05. (b) Flow cytometry was used to detect the apoptosis rate of HCC827 cell spheres treated with 200 *μ*M CUR, 5 mg/mL CBP, and their combination for 48 h. (c) The effect of 200 *μ*M CUR on the cell pelletization ability of HCC827 cell spheres treated for 14 days was detected under a phase contrast microscope. ^A^Compared with the control group, *P* < 0.01.

CCK-8 showed that the cell inhibition rates of 5 mg/mL CBP group, 200 *μ*M CUR group, and 5 mg/mL CBP+200 *μ*M CUR group were significantly higher than those of control group at 24 h, 36 h, and 48 h (Figure 3(a)) (*P* < 0.05). In addition, the cell inhibition rate of the 5 mg/mL CBP+200 *μ*M CUR group was higher than that of the 5 mg/mL CBP group and 200 *μ*M CUR group at 24 h, 36 h, and 48 h (*P* < 0.05). Flow cytometry showed that the apoptosis rate of 5 mg/mL CBP+200 *μ*M CUR group, 5 mg/mL CBP group, and 200 *μ*M CUR group was significantly higher than that of control group (Figure 3(b)). In cell pelletization experiment, we found that after 14 days of treatment with 200 *μ*M CUR, the cell connection in the HCC827cell spheres is loose, and the refraction of the cell is weakened, and the number of cell spheres were also decreased significantly (Figure 3(c)). These studies indicated that CUR could effectively inhibit the proliferation, apoptosis, and cell pelletization ability of HCC827 cell spheres.

### 3.4. Effects of CUR Intervention on SOX2, ABCG2, and *β*-Catenin Expression and *β*-Catenin Promoter Activity in HCC827 Cell Spheres

**Figure 4:**
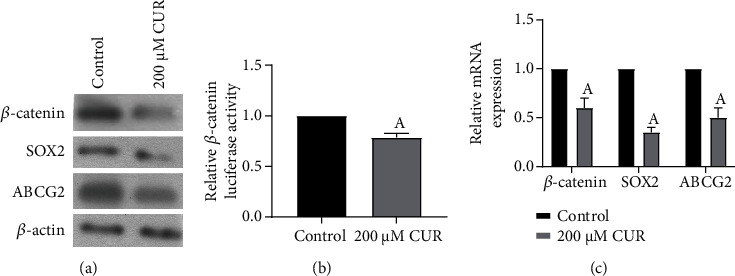
Effects of CUR intervention on SOX2, ABCG2, and *β*-catenin expression and *β*-catenin promoter activity in HCC827 cell spheres. (a) Western blot was used to detect the protein levels of SOX2, ABCG2, and *β*-catenin in HCC827 cell spheres treated with 200 *μ*M CUR for 48 h. (b) Changes of *β*-catenin promoter activity in HCC827 cell spheres treated with 200 *μ*M CUR for 48 h were detected by luciferase reporter gene. ^A^Compared with the control group, *P* < 0.05. (c) qPCR was used to detect the mRNA levels of SOX2, ABCG2, and *β*-catenin in HCC827 cell spheres treated with 200 *μ*M CUR for 48 h. ^A^Compared with the control group, *P* < 0.05.

Western blot and qPCR showed that the protein and mRNA levels of *β*-catenin, SOX2, and ABCG2 were significantly decreased after HCC827 cell spheres were treated with 200 *μ*M CUR for 48 h (Figures 4(a) and 4(c)) (*P* < 0.05). In addition, luciferase reporter gene assay showed that 200 *μ*M CUR significantly inhibited *β*-catenin promoter activity (Figure 4(b)) (*P* < 0.05). These findings suggested that CUR downregulated the expression of *β*-catenin and its downstream SOX2 and ABCG2 by inhibiting *β*-catenin promoter activity.

### 3.5. Overexpression of *β*-Catenin Attenuates the Inhibitory Effect of CUR on HCC827 Cell Globules

**Figure 5:**
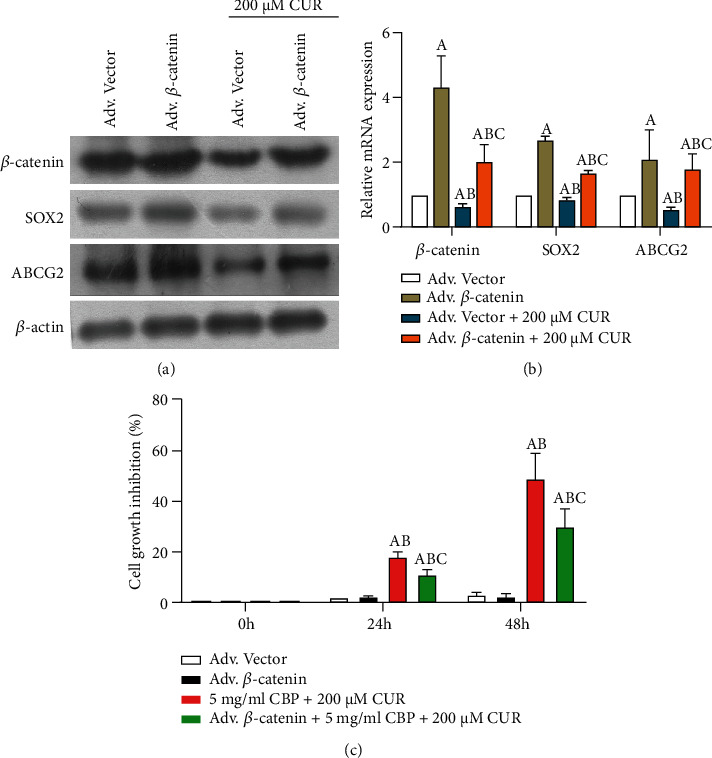
Overexpression of *β*-catenin attenuates the inhibitory effect of CUR on HCC827 cell spheres. (a, b) The protein and mRNA levels of SOX2, ABCG2, and *β*-catenin were detected by Western blot and qPCR. HCC827 cell spheres were treated with 200 *μ*M CUR for 48 h after 12 h of infection with Adv. *β*-catenin adenovirus. ^A^Compared with Adv. Vector, *P* < 0.05. ^B^Compared with Adv. *β*-catenin group, *P* < 0.05. ^C^Compared with Adv. Vector+200 *μ*M CUR group, *P* < 0.05. (c) CCK-8 detected the change of cell spheres inhibition rate after different treatments. 5 mg/mL CBP+200 *μ*M CUR combined treatment group: HCC827 cell spheres were treated with 5 mg/mL CBP+200 *μ*M CUR. Adv *β*-catenin+5 mg/mL CBP+200 *μ*M CUR combined treatment group: *β*-catenin adenovirus HCC827 cell spheres were infected with Adv. *β*-catenin for 12 h. HCC827 cell spheres were treated with 5 mg/mL CBP+200 *μ*M CUR. ^A^Compared with Adv. Vector, *P* < 0.05. ^B^Compared with Adv. *β*-catenin group, *P* < 0.05. ^C^Compared with 5 mg/mL CBP+200 *μ*M CUR combined treatment group, *P* < 0.05.

After we overexpressed *β*-catenin using adenovirus, western blot and qPCR showed that the protein and mRNA levels of *β*-catenin, SOX2, and ABCG2 were significantly increased (Figures 5(a) and 5(b)) (*P* < 0.05). The protein and mRNA expressions of *β*-catenin, SOX2, and ABCG2 were significantly inhibited after 200 *μ*M CUR treatment of *β*-catenin expressing HCC827 cell spheres (*P* < 0.05). CCK-8 was used to detect the change of cell spheres inhibition rate. The results showed that overexpression of *β*-catenin significantly reduced the inhibitory effect of 5 mg/mL CBP and 200 *μ*M CUR on the proliferation of HCC827 cell spheres.

## 4. Discussion

In this study, LCSC-rich HCC827 cell spheres were successfully induced, which could tolerate CBP killing. Application of CUR can significantly reduce the activity of GST in HCC827 cell spheres. The upregulation of ROS level inhibited the expression of *β*-catenin and SOX2 and the activity of *β*-catenin promoter, which inhibited the proliferation and cells pelletization ability of HCC827 cell spheres and promoted cell apoptosis. In addition, CUR inhibited the expression of drug resistance related protein ABCG2. Therefore, CUR combined with CBP can kill HCC827 cell spheres more effectively than CUR alone, and the upregulation of *β*-catenin expression can partially reverse the inhibitory effect of CUR combined with CBP on HCC827 cell spheres. These studies suggest that CUR can effectively kill LCSC-rich HCC827 cell spheres, possibly through upregulation of ROS and downregulation of *β*-catenin, SOX2, and ABCG2.

CSCs play a variety of roles in tumor genesis and progression. Previous studies have shown, according to certain stem cell markers [17–19], magnetic activated cell sorting (MACS) or fluorescence-activated cell sorter (FACS) isolation of CSCs from multiple lung cancer cell lines and lung cancer. These CSCs can form cell spheres in serum-free medium (SFM). Since parental CSCs in tumors only pass on their stem cell properties to their offspring, tumor cells can form clones of different forms in vitro: tight clones (holoclones) are due to the composition of CSCs, while loose clones (paraclone) do not have the characteristics of CSCs [20, 21]. Therefore, in our previous study and this study, selected compact clones were used for serum-free culture to obtain HCC827 cell spheres [22]. Compared with HCC827 cells, the protein expression levels of stem cell markers CD133, SOX2, and EpCAM were significantly increased in HCC827 cell spheres. Moreover, HCC827 cells upregulated the expression of drug resistance-related gene ABCG2 and could tolerate the killing effect of CBP. The above study indicated that we successfully isolated and cultured HCC827 cell spheres rich in tumor stem cells, which laid a foundation for further research.

Recent studies have shown that CUR can not only directly kill tumor cells but also enhance tyrosine kinase inhibitors (TKIs) and carboplatin against EGFR-mutated non-small-cell lung cancer [23]. Our study also found that CUR not only effectively killed HCC827 cell spheres but also reversed the natural resistance of HCC827 cell spheres to CBP, enabling CBP to kill HCC827 cell spheres effectively. In addition, CUR also significantly inhibited the formation of HCC827 cell spheres. These studies indicate that CUR is an effective drug to inhibit tumor stem cells. Studies have shown that inhibition of GST is one of the important pathways of CUR. GST is usually highly expressed in tumor cells, which plays a role in cellular detoxification and resistance to drug killing by reducing the formation of GSH and binding to electron-philic components. In this study, CUR significantly inhibited the activity of GST in HCC827 cell spheres, which may weaken the resistance of HCC827 cell spheres to CBP and enhance the cytotoxicity of CBP. In addition, CUR was found to have high antioxidant activity and anticancer properties, which may be caused by upregulation of ROS levels in cells and inhibition of GST activity by CUR [24, 25].

A large number of studies have shown that Wnt signaling pathway is abnormally activated in a variety of tumors and is involved in the occurrence, progression, and metastasis of tumors [26, 27]. Wnt signaling also plays a key role in tumor stem cells: activation of Wnt/*β*-catenin signaling pathway can promote the tumor stem cell characteristics of ovarian cancer cells and esophageal cancer cells [28, 29], possibly through upregulation of tumor stem cell markers Nanog, OCT-4, and SOX2 [30, 31]. It has been suggested that CUR inhibits the Wnt/*β*-catenin signaling pathway by directly inhibiting Wnt5a or accelerating the degradation of *β*-catenin by reducing its stability [32]. Our study found that CUR not only inhibited *β*-catenin expression but also significantly inhibited the activity of *β*-catenin promoter and the dryness of HCC827 cell spheres (downregulation of SOX2 and inhibition of pelletizing ability). However, adenovirus overexpression of *β*-catenin significantly reduced the inhibitory effect of CUR on HCC827 cell spheres proliferation and downregulation of SOX2 and ABCG2 downstream of *β*-catenin. These results suggest that *β*-catenin is a key molecule in the maintenance of tumor stem cell properties in HCC827 cell spheres and a key target of CUR. CUR may inhibit *β*-catenin by downregulating protein stability and inhibiting promoter activity.

This study confirmed that CUR can effectively inhibit the proliferation and dryness of LCSC-rich HCC827 cell spheres and induce their apoptosis. At the same time, CUR can reverse the resistance of HCC827 cell spheres to CBP, and the combination of CBP and CUR can kill HCC827 cell spheres more effectively. CUR plays an important role by inhibiting GST, promoting ROS production, and inhibiting *β*-catenin and its downstream SOX2 and ABCG2. These results indicate that CUR is an effective drug against lung cancer and lung cancer stem cells, which is expected to be applied in clinic. The results of this study remain at the cellular level for the time being, and future animal experiments are needed to further verify the in vivo efficacy of curcumin and carboplatin.

## Figures and Tables

**Table 1:** Primers, amplification conditions, and product size of target genes.

Gene	Primer sequence (5′⟶3′)	Product length	Annealing temperature
SOX2	F: 5′-ATCACCCACAGCAAATGACA-3′ R: 5′-CAAAGCTCCTACCGTACCACTA-3′	245 bp	60°C
*β*-Catenin	F: 5′-GCGCCATTTTAAGCCTCTCG-3′ R: 5′-AAATACCCTCAGGGGAACAGG-3′	183 bp	60°C
ABCG2	F: 5′-GAACCCAAGGAGATAGGAGA-3′ R: 5′- CTAGACAGACTTCAACCAGG -3′	225 bp	60°C
*β*-Actin	F: 5′-CCTGTACGCCAACACAGTGC-3′ R: 5′-ATACTCCTGCTTGCTGATCC-3′	211 bp	60°C

## Data Availability

The data used to support the findings of this study are included within the article.
